# Impact of the Location of CpG Methylation within the *GSTP1* Gene on Its Specificity as a DNA Marker for Hepatocellular Carcinoma

**DOI:** 10.1371/journal.pone.0035789

**Published:** 2012-04-20

**Authors:** Surbhi Jain, Sitong Chen, Kung-Chao Chang, Yih-Jyh Lin, Chi-Tan Hu, Batbold Boldbaatar, James P. Hamilton, Selena Y. Lin, Ting-Tsung Chang, Shun-Hua Chen, Wei Song, Stephen J. Meltzer, Timothy M. Block, Ying-Hsiu Su

**Affiliations:** 1 Department of Microbiology and Immunology, Drexel University College of Medicine, Philadelphia, Pennsylvania, United States of America; 2 Department of Pathology, College of Medicine, National Cheng Kung University, Tainan, Taiwan, Republic of China; 3 Department of Surgery, National Cheng Kung University Hospital, Tainan, Taiwan, Republic of China; 4 Department of Medicine, Buddhist Tzu Chi General Hospital and Tzu Chi University, Hualien, Taiwan, Republic of China; 5 Division of Gastroenterology and Hepatology, Department of Medicine, The Johns Hopkins University School of Medicine, Baltimore, Maryland, United States of America; 6 Department of Medicine, College of Medicine, National Cheng Kung University, Tainan, Taiwan, Republic of China; 7 Department of Microbiology, Medical College, National Cheng Kung University, Tainan, Taiwan, Republic of China; 8 JBS Science Inc., Doylestown, Pennsylvania, United States of America; 9 Department of Oncology, The Sidney Kimmel Comprehensive Cancer Center, Baltimore, Maryland, United States of America; National Cancer Institute, United States of America

## Abstract

Hypermethylation of the glutathione S-transferase π 1 (*GSTP1*) gene promoter region has been reported to be a potential biomarker to distinguish hepatocellular carcinoma (HCC) from other liver diseases. However, reports regarding how specific a marker it is have ranged from 100% to 0%. We hypothesized that, to a large extent, the variation of specificity depends on the location of the CpG sites analyzed. To test this hypothesis, we compared the methylation status of the *GSTP1* promoter region of the DNA isolated from HCC, cirrhosis, hepatitis, and normal liver tissues by bisulfite–PCR sequencing. We found that the 5′ region of the position −48 nt from the transcription start site of the *GSTP1* gene is selectively methylated in HCC, whereas the 3′ region is methylated in all liver tissues examined, including normal liver and the HCC tissue. Interestingly, when DNA derived from fetal liver and 11 nonhepatic normal tissue was also examined by bisulfite-PCR sequencing, we found that methylation of the 3′ region of the promoter appeared to be liver-specific. A methylation-specific PCR assay targeting the 5′ region of the promoter was developed and used to quantify the methylated *GSTP1* gene in various diseased liver tissues including HCC. When we used an assay targeting the 3′ region, we found that the methylation of the 5′-end of the *GSTP1* promoter was significantly more specific than that of the 3′-end (97.1% vs. 60%, p<0.0001 by Fisher's exact test) for distinguishing HCC (n = 120) from hepatitis (n = 35) and cirrhosis (n = 35). Encouragingly, 33.8% of the AFP-negative HCC contained the methylated *GSTP1* gene. This study clearly demonstrates the importance of the location of CpG site methylation for HCC specificity and how liver-specific DNA methylation should be considered when an epigenetic DNA marker is studied for detection of HCC.

## Introduction

Hepatocellular carcinoma (HCC) is the third or fourth leading cause of cancer deaths in the world, the second fastest growing cancer, and is now one of the top 10 causes of cancer deaths in the United States [1]. It is usually an aggressive malignancy with a 5-year survival rate of as low as 14% [1], [2]. The 5-year survival rate of patients with early-stage HCC is 26% but only 2% when it is found after metastasis to distant organs. Therefore, early detection is critical for effective treatment of HCC. The current circulating marker, alpha-fetoprotein (AFP), and its fucosylated glycoform, L3, are of limited value, with a sensitivity of only 40% to 60% [3]. Thus, there is an urgent need for a better marker or panel of markers for the early detection of HCC.

The development of HCC, as with other solid tumors, is believed to require the dysregulation of at least three biochemical pathways (proliferation, cell cycle, apoptosis/cell survival) within the cell [4], [5], [6]. In addition to genetic mutations, the aberrant methylation of tumor suppressors plays an important role throughout the process of HCC carcinogenesis. Due to the high heterogeneity of HCC, a panel of markers may be needed to provide sufficient sensitivity for its detection, and combinations of markers for HCC detection and management have been suggested by several groups [7], [8], [9], [10], [11], [12], [13], [14], [15].

For biomarkers to be most useful for early detection or screening, one would need to be able to detect the markers without biopsy, such as in the circulation. In the course of assembling a panel of HCC markers that have been detected in the blood of patients with HCC for the development of a potential screening test for the early detection of HCC, we noticed that methylated glutathione S-transferase π 1 (m*GSTP1*), one of the extensively studied DNA markers, was detected in the circulation of patients with HCC [16], [17], [18], [19]. However, variability in the HCC specificity of *mGSTP1* has been reported in a number of previously published studies, examples of which are shown in Table 1.

**Table 1:** Examples of the variable specificities of methylated *GSTP1* genes for HCC reported in previous studies.

Study	*GSTP1* Methylation # methylated/total (%)		CpG Sites Studied^1^	Sensitivity (%)	Specificity (%)	Method Analyzed
	HCC	Normal liver				
1 [14]	18/34 (53)	0/16 (0)	−27 to −7	53	100	MethyLight
2 [7]	46/60 (77)	0/20 (0)	−19 to −6	77	100	MSP
3 [13]	28/51 (54)	3/22 (13.7)	−19 to −7	54	86.4	MSP
4 [42]	16/20 (80)	3/3 (100)	−11 to +4	80	0	Pyrosequencing
5 [15]	24/40 (60)	12/25 (48)	−4 to +7	60	48	MethyLight

1 The CpG site number is referred to the transcription start site.

HCC, hepatocellular carcinoma; MSP, methylation-specific PCR.

The *GSTP1* gene encodes glutathione S-transferase π, which protects normal hepatocytes against a number of mutation-inducing processes, such as reactive oxygen species linked with chronic hepatic inflammation and reactive electrophilic compounds linked with the hepatic metabolism of dietary and other carcinogens [20], [21], [22], [23]. Hypermethylation of its promoter region has been shown to suppress the expression of the *GSTP1* gene [24], [25], [26]. Thus, the hypermethylation of the promoter of the *GSTP1* gene has been associated with various cancers, including HCC [9], [10], [11], [13], [14], [24], [27], [28], [29], [30], [31].

Recent evidence, as reviewed by van Vlodrop, has implicated the impact of the location of aberrant CpG dinucleotide methylation on gene expression and on its clinical value in cancer [32]. This work suggests that the current data on hypermethylation markers require a more comprehensive and critical evaluation prior to their implementation in clinical practice. Our recent study of the methylation of the adenomatous polyposis coli (*APC*) gene also suggested that not only the location of CpG methylation but also the strand of DNA analyzed could have an impact on the specificity of the methylated *APC* gene as a marker to distinguish HCC from other liver diseases [33]. In this study, the *APC* gene was found to be methylated preferentially on the antisense strand of the DNA in normal liver. Thus methylation of the CpG sites only on the sense strand of the *APC* gene is specific for HCC. Interestingly, Millar et al. [34] studied the association of aberrant methylation of the *GSTP1* with prostate cancer and noticed that DNA methylation of the sense strand of the *GSTP1* gene in normal liver appeared to be different from that from nine other organs examined. We thus hypothesized that the variability of HCC specificity of the *mGSTP1* gene observed in previous studies could be due to the region of the promoter analyzed.

Patients with hepatitis or cirrhosis are known to be at high risk for HCC. Thus, a biomarker for HCC screening that distinguishes HCC from hepatitis and cirrhosis would be of particular usefulness. Although several studies have suggested a role for the *mGSTP1* promoter as a biomarker for HCC detection, to our knowledge, the methylation status of the *GSTP1* promoter has not been analyzed by bisulfite sequencing, which is used to analyze every CpG site in the region examined, across the spectrum of normal liver, hepatitis, cirrhosis, and HCC. In this study, we performed a comprehensive detailed methylation analysis using bisulfite sequencing for both sense and antisense strands in normal and diseased liver tissue (hepatitis, cirrhosis, and matched HCC and adjacent non-HCC) in order to identify HCC-specific CpG sites to develop an assay in the future study that would detect *mGSTP1* specifically in the circulation. We demonstrated that only methylation of a subset of the CpG sites in the *GSTP1* promoter, the 5′ end of the position −48 nucleotide [nt] relative to the transcription start site, was methylated specifically in HCC and also confirmed that the methylation of the CpG sites of the *GSTP1* gene at the 3′-end promoter region occurs in the normal liver and appears to be liver specific compared to DNA isolated from 12 other normal tissues. Furthermore, after comparing the matched adjacent non-HCC tissues, we suggest a cancer field effect of *GSTP1* methylation.

## Results

### DNA methylation profiles of the promoter region of the *GSTP1* gene in normal liver and diseased liver tissues

**Figure 1:**
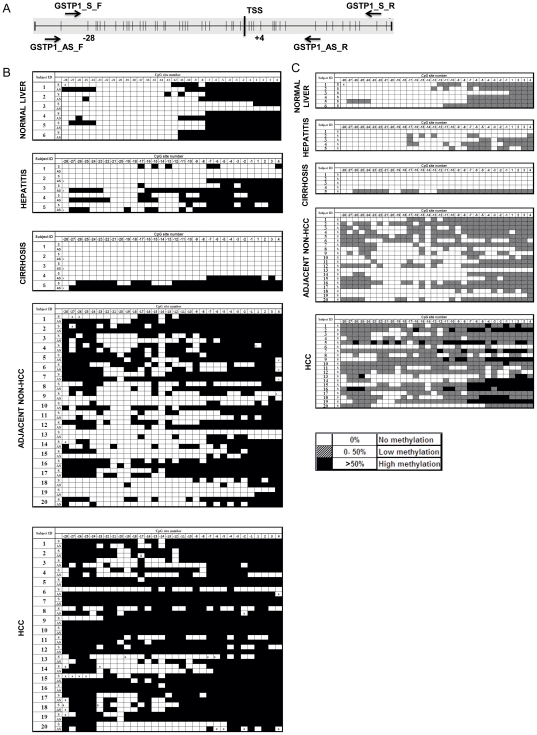
Methylation profiles of the sense and antisense strands of the *GSTP1* gene by BSP sequencing of DNA isolated from normal liver and diseased liver tissues. (A) Diagram of the locations of bisulfite sequencing primers and the CpG sites, indicated by vertical bars, in the promoter and the first exon regions of the *GSTP1* gene (Genbank accession #M24485, nt. 999–1387). The transcription start site (TSS) is also indicated. The CpG sites are bracketed by the bisulfite sequencing primers for the forward (F) and reverse (R) sense strands (GSTP1_S_F and GSTP1_S_R) and the antisense strands (GSTP1_AS_F and GSTP1_AS_R). (B) Methylation status of each CpG site in both sense (S) and antisense (AS) strands of the promoter and the first exon regions of the *GSTP1* gene from −28 to +4 on the basis of the sense strand 5′ to 3′ direction relative to TSS in hepatocellular carcinoma (HCC, n = 20) tissue, matched adjacent non-HCC liver tissue (Adj Non-HCC, n = 20), and normal (n = 6), hepatitis (n = 5), and cirrhosis (n = 5) tissues. The filled boxes indicate methylation detected and open boxes indicate no methylation detected. (C) Analysis of the extent of methylation at each CpG site of the sense strand *GSTP1* gene by BS-PCR sequencing of DNA isolated from normal liver and diseased liver tissues. CpG site locations, BS-PCR sequencing assay, and DNA samples are the same as in panel B. The filled boxes indicate a high level of methylation detected (more than 50%); hatched boxes indicate a low level of methylation detected (50% or less); and open boxes indicate no methylation detected.

To test the hypothesis that the variable specificities of the methylated *GSTP1 (mGSTP1)* gene for HCC reported in previous studies could be due to the location within the gene analyzed, a comprehensive survey of the promoter region using a bisulfite-specific PCR (BS-PCR) assay followed by DNA sequencing was performed. Because we previously demonstrated that methylation could occur in a strand-biased manner, such as the antisense strand-biased methylation of the *APC* gene in normal liver [33], it was of interest to determine whether the methylation of the *GSTP1* promoter in liver was also DNA strand-biased. Therefore, we examined the methylation profile of both the sense and the antisense strands of the *GSTP1* promoter region. Bisulfite-specific primers (BSP) for both the sense (*GSTP1*_BSP_S) and antisense strands (*GSTP1*_BSP_AS) of the promoter region were designed to include most of the CpG sites that were analyzed in the previous studies listed in Table 1. Figure 1A shows CpG sites (vertical bars) in the promoter and first exon regions of the *GSTP1* gene, along with locations of BSP primers (primer sequences are listed in Table S1). All 32 CpG sites within the region studied were numbered from −28 to +4 relative to the transcription start site in the 5′ to 3′ direction.

To determine the assay sensitivity of each BS-PCR sequencing reaction detecting the methylated CpG, we performed a reconstitution experiment using a set of standards with varying proportions of methylated DNA as indicated in Figure S1. We found that, although the BS-PCR sequencing assays for both the sense and antisense strands were able to consistently detect methylated CpG in samples containing 10% methylated DNA present in an excess of 90% unmethylated DNA, the resolution of the extent of methylation was higher for the sense assay than for the antisense assay. When a sample containing 10% methylated DNA was tested in the reconstitution experiments, the sequencing chromatograph generated by the antisense BS-PCR sequencing assay showed only the “C” peak, whereas a mix of “C” and “T” peaks were observed with the sense BS-PCR sequencing assay.

With the sensitivity of the assay determined, we performed BS-PCR sequencing in normal (n = 6) and diseased liver including hepatitis (n = 5), cirrhosis (n = 5), and HCC and matched non-HCC tissues (n = 20). The clinicopathological information for the study subjects is described in Table 2. To control for the efficiency of the bisulfite conversion, we determined the percentage of cytosine-to-thymine conversions that occurred in non-CpG cytosines within the analyzed region after DNA sequencing of BSP product from each sample. Only samples yielding a cytosine-to-thymine conversion rate higher than 95% for these non-CpG Cs were analyzed further.

**Table 2:** Summary of clinicopathological characteristics of the tissues analyzed by BSP sequencing.

Characteristic	Normal^1^ (n = 6)	Hepatitis (n = 5)	Cirrhosis (n = 5)	HCC (n = 20)
**Mean age ± SD, years**	64±7.5	59.8±10.3	60.6±14.9	60.2±12.4
**Male/female**	4/2	4/1	2/3	11/9
**HBV/HCV/other**	0/0/0	2/3/1	2/2/1	7/9/5
**Stage 1/2/3/4/unknown**	-	-	-	10/7/1/1/1
**Grade 1/2/3/unknown**	-	-	-	3/11/5/1
**Mean size of tumor ± SD, cm**	-	-	-	5.27±2.67
**AFP levels, ng/mL, ≤20/>20/unknown**	-	-	-	8/11/1

1 4/6 of the 6 normal livers are “normal” liver tissues with concomitant cholangiocarcinoma.

AFP, alpha-fetoprotein; HBV, hepatitis B virus; HCC, hepatocellular carcinoma; HCV, hepatitis C virus; SD, standard deviation.

Due to the different resolutions obtained using the sense and antisense BS-PCR sequencing assays, we constructed the methylation profile of the *GSTP1* promoter for both sense and antisense strands using a dichotomized variable: methylation detected or undetected, as shown in Figure 1B. As suggested by Millar et al. [34], when we studied the methylation status of the sense strand of the *GSTP1* promoter, we found that the CpG sites of the sense strand of the *GSTP1* DNA from −7 to +4 were methylated in normal liver tissue and that the methylation was mostly symmetrical for both sense and antisense strands of the DNA. In contrast, most of the first 21 CpG sites (−28 to −8) were not methylated on either the sense or the antisense strand of DNA (Fig. 1B), although minimal nonsymmetrical methylation was observed in some samples. We thus divided the region examined into the 5′-end of the *GSTP1* promoter, which included CpG sites −28 to −8, or those upstream from the nt position −48 from the transcription start site, and the 3′-end of the promoter, which included CpG sites −7 to +4, or those downstream from the nt position −48 in this study. Interestingly, the majority of the CpG sites in the 5′-end of the promoter region did not appear to be methylated in either hepatitis or cirrhotic liver but were methylated in HCC and even adjacent non-HCC liver tissues, as shown in Figure 1B.

Because the variable specificities of the *mGSTP1* gene for HCC reported in previous studies were all obtained from an analysis of the sense strand, to test our hypothesis, we compared the specificities of the 3′-end and 5′-end of the *GSTP1* gene only on the sense strand and categorized the extent of methylation obtained from results from the reconstitution experiments (supplemental Figure 1) into three categories: no methylation detected (0% detectable methylated DNA); low level of methylation detected (both “C” and “T” peaks detected, 0–50% methylated DNA), and a high level of methylation detected (“C” peak only, >50% methylated DNA) as shown in Figure 1C. We calculated the percent of the methylated CpG sites detected per total CpG sites analyzed for both the 3′-end and 5′-end regions, as shown in Table 3. For the region of the 5′-end promoter, low levels of methylation were detected in normal (11.1%), hepatitis (8.6%), cirrhosis (13.3%), adjacent non-HCC (36.9%), and HCC (59.8%) tissue; however, only the CpG sites in the HCC tissue contained a high level of methylation (5.2%). The mean level of methylation detected (low+high), together with the corresponding standard deviation, was calculated for each tissue group. To see if the specificity of *mGSTP1* for HCC differed between the 3′-end and the 5′-end, we determined the p value of HCC compared with those of all liver tissues from non-HCC patients (cirrhosis+hepatitis+normal liver) for each region. As indicated by Fig. 1B, an increase in the amount of methylation was observed in adjacent non-HCC tissue compared to that of normal liver. Of interest, we also determined the p value of the HCC tissue compared with all non-HCC tissues including adjacent non-HCC tissue, cirrhosis, hepatitis, and normal liver. As shown in Table 3, the level of methylation was significantly higher (p<0.0001: by Student's *t* test) in the HCC compared to the non-HCC groups (both with and without the adjacent non-HCC tissue). In contrast, a similar analysis was performed for the region of the 3′-end promoter; no significant difference was obtained (p>0.05) for the amount of methylation detected in the HCC group compared with the non-HCC groups (with or without adjacent non-HCC). These data suggested that the methylation of the 5′-end promoter, compared to the 3′-end, should have more discriminatory power (specificity) to distinguish HCC from hepatitis and cirrhosis.

**Table 3:** Comparison of the extent of CpG methylation of the GSTP1 sense strand promoter region among various normal and diseased liver tissues^1^.

Promoter Region^2^	5′-end					3′-end				
Tissue type	Normal (n = 6)	Hepatitis (n = 5)	Cirrhosis (n = 5)	Adjacent non-HCC (n = 20)	HCC (n = 20)	Normal (n = 6)	Hepatitis (n = 5)	Cirrhosis (n = 5)	Adjacent non-HCC (n = 20)	HCC (n = 20)
**No methylation, %** 3	88.9	91.4	86.7	63.1	35.0	12.1	49.1	81.5	30.0	15.5
**Low methylation, %** 3	11.1	8.6	13.3	36.9	59.8	87.9	50.9	18.5	70.0	47.7
**High methylation. %** 3	0.0	0.0	0.0	0.0	5.2	0.0	0.0	0.0	0.0	36.8
**Mean methylation detected ± standard deviation, %** 3	11±9	9±8	13±30	37±28	65±28	88±25	51±36	18±41	37±20	44±14
**p value** 4	HCC vs normal+hepatitis+cirrhosis = 8.5E-06					HCC vs normal+hepatitis+cirrhosis = 0.959				
	HCC vs normal+hepatitis+cirrhosis+adjacent non-HCC = 4.4E-08					HCC vs normal+hepatitis+cirrhosis+adjacent non-HCC = 0.376				

1 The extent of methylation at each CpG site was analyzed as in Figure 1C: no methylation (no methylation detected); low methylation (50% or less); high methylation (more than 50%).

2 The CpG sites included in the 5′-end and the 3′-end are described in the text.

3 The percent of CpG methylation was calculated as the number of methylated CpG sites per category/total CpG sites analyzed×100%.

4 p value was determined using Student's *t* test.

HCC, hepatocellular carcinoma.

### Methylation profile of the promoter region of the *GSTP1* gene in non-liver normal tissues

**Figure 2:**
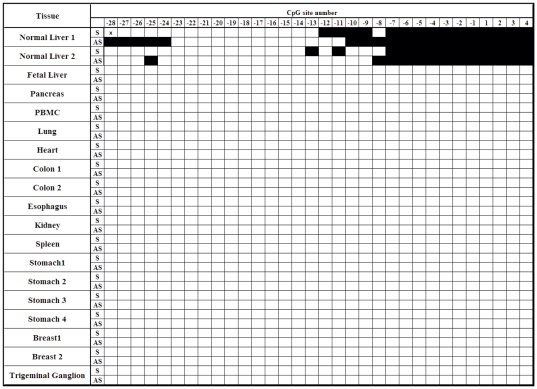
Methylation status of the sense (S) and antisense (AS) strands of the promoter and the first exon region of the *GSTP1 gene* (Genebank accession #M24485, nt. 999–1387) of the DNA isolated from normal adult livers, fetal liver, and normal nonliver tissues. The open boxes indicate unmethylated CpG sites; the filled boxes indicate methylation detected.

Our recent study showed the presence of liver-specific, antisense-biased CpG methylation in the promoter region of the tumor suppressor *APC* gene [33]. Millar et al. [34] suggested that, on analysis of the sense strand of the DNA from eight different tissue samples, methylation of the 3′-end of the promoter region of the *GSTP1* gene was found only in normal liver [34]. Thus, to determine if the methylation pattern of the *GSTP1* promoter seen in normal liver tissue is liver-specific, we examined the methylation profile of both the sense and the antisense strands of the *GSTP1* promoter region in 11 other non-liver normal tissues including pancreas, peripheral blood monocytes, lung, heart, colon, esophagus, kidney, spleen, stomach, breast, and trigeminal ganglion and 1 fetal liver, as shown in Figure 2. As a reference of normal adult liver, we included the methylation profiles from two normal livers from normal healthy subjects in Figure 2. All 11 nonliver and 1 fetal liver normal tissue samples showed no detectable methylation in either the 5′-end or the 3′-end of the promoter regions, indicating that methylation of the 3′-end promoter region of the *GSTP1* appears to be liver-specific.

### Evaluation of the specificity of the *mGSTP1* promoter in distinguishing HCC from other liver diseases by the methylation-specific PCR assay (MSP)

**Figure 3:**
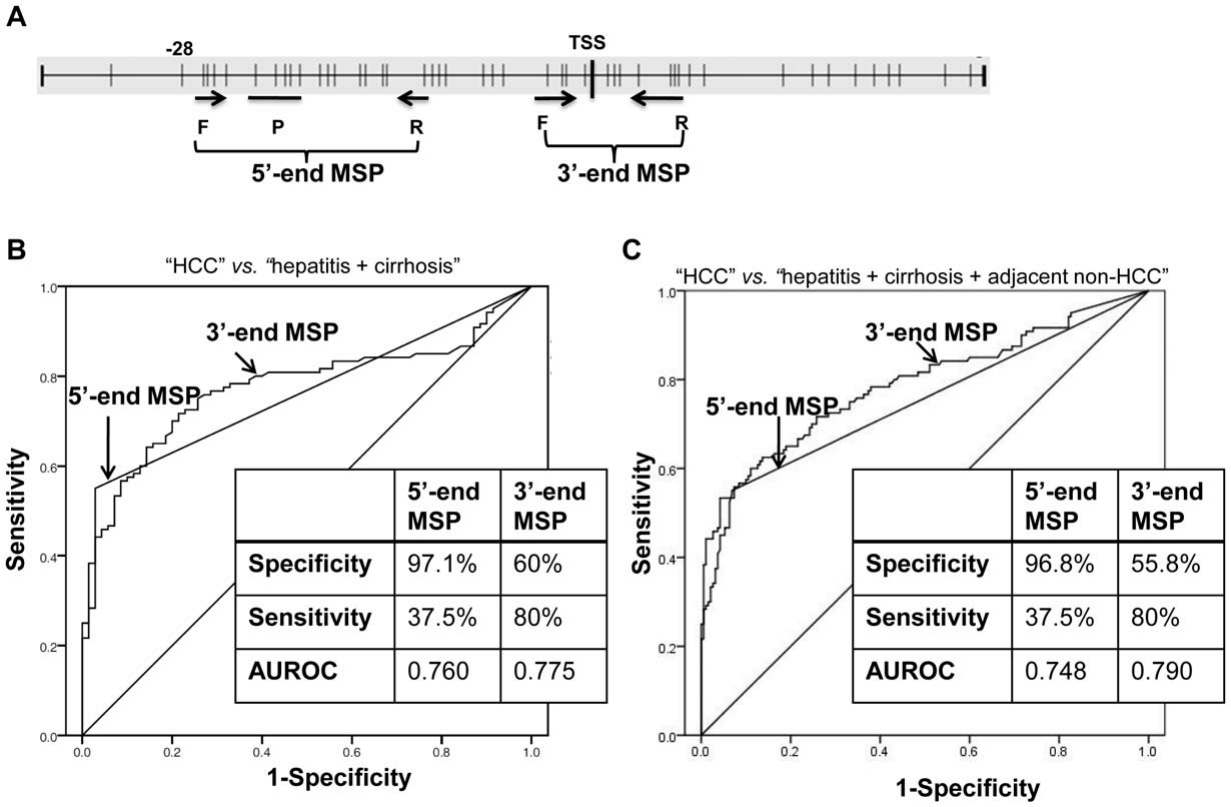
Comparison of the specificity of the 5′-end and the 3-end of the *mGSTP1* as a biomarker to distinguish HCC samples from tissue samples of other liver diseases, as determined by MSP assays. (A) Locations of forward (F) and reverse (R) primers and TaqMan probe (P) of the 5′-end MSP (5′-MSP) (including the TaqMan probe) and 3′-end MSP (3′-MSP) SybrGreen assays. The CpG sites (vertical bars) and the transcription start site (TSS) are indicated. Receiver operating characteristic (ROC) curves of the methylated *GSTP1* gene as a marker to discriminate HCC (n = 120) from non-HCC liver tissues including hepatitis (n = 35) and cirrhosis (n = 35) (B), or hepatitis, cirrhosis, and adjacent non-HCC (C), generated by 5′-end MSP and 3′-end MSP assays, respectively, as indicated. The amount of methylated DNA was the average of two duplicate MSP assays as detailed in Materials and Methods. The area under the curve of each ROC (AUROC) curve and the specificity and sensitivity determined by the cutoff of 10 copies per input of 300 copies of DNA are shown in the inserted table. Note that the CpG sites included in each primer and probe are as follows; 5′-end MSP (F: −27 to −24; P: −23 to −19; R: −11 to −10) and 3′-end MSP (F: −4 to −2; R: +4 to +7).

Bisulfite sequencing is a method used to analyze the methylation status of every CpG site in the region of interest, but it is labor intensive to analyze a large panel of samples representing the different stages of disease progression to HCC. To confirm our results from the BS-PCR sequencing, showing that the methylation of the 5′-end region of the *GSTP1* promoter is more specific to HCC than that of the 3′-end region in a large sample of tissues, we developed an MSP assay targeting the CpG sites that showed higher HCC specificity when analyzed by BS-PCR sequencing (Fig. 1B) in the 5′-end promoter region. In this MSP assay, the CpG sites included in the forward primer, the TaqMan probe, and the reverse primers are −27 to −24, −23 to −19, and −11 and −10, respectively (Fig. 3A). The sequences of the primers and probe and the PCR conditions are described in Table S1. Using this assay, we were able to quantify methylated DNA with an assay sensitivity of 10 copies per reaction, as shown in Figure S2A. As a comparison, we used an MSP assay reported previously [15] to target the 3′-end of the *GSTP1* promoter region. This assay has a sensitivity and linear range for quantifying the *mGSTP1* gene comparable to the 5′-end MSP assay that we developed, as shown in Figure S2B.

Next, we compared the performance of *mGSTP1* in the 5′-end MSP assay with that in the 3′-end MSP assay [15] in distinguishing HCC from hepatitis and cirrhosis. The DNA isolated from hepatitis (n = 35), cirrhosis (n = 35), adjacent non-HCC (n = 120), and HCC samples (n = 120) was treated with bisulfite and quantified by the BS-actin PCR assay as described previously [33]. The clinicopathological characteristics of the study subjects are shown in Table 4, which includes the study subjects used in the BS-PCR sequencing assays. The amount of methylated *GSTP1* promoter was determined for each segment of BS-converted DNA by MSP assays in duplicate, and the receiver operating characteristic (ROC) curves were constructed accordingly to evaluate the performance of the *mGSTP1* promoter as a biomarker to distinguish HCC from non-HCC including hepatitis and cirrhosis (Fig. 3B). We also constructed and compared ROC curves for HCC tissue and for non-HCC tissues including adjacent non-HCC tissues (Fig. 3C). The specificity and sensitivity were calculated on the basis of the cutoff value of 10 copies since that is the limit of the quantification for both 3′-end and 5′-end MSP assays. As listed in the table inset in Figure 3B, as a biomarker to distinguish HCC from non-HCC liver diseases, the methylation of the 5′-end of the *GSTP1* promoter was significantly more specific than that of the 3′-end (97.1% vs. 60%, p<0.0001 by Fisher's exact test), although the 5′-end was less sensitive than the 3′-end (37.5% vs. 80%), and the AUROC curves for both regions were similar (0.760 and 0.775). We obtained a similar result, i.e., significantly more specific methylation at the 5′-end of the *GSTP1* promoter than at the 3′-end (96.8% vs. 55.8%, p<0.0001 by Fisher's exact test), when the comparison included the adjacent non-HCC tissues (Fig. 3C).

**Table 4:** Summary of clinicopathological characteristics of the tissues analyzed using the MSP assays.

Characteristic	Normal^1^ (n = 6)	Hepatitis (n = 35)	Cirrhosis (n = 35)	HCC (n = 120)	P value
**Mean age ± SD, years**	64±7.5	55±11.62	56±13.8	60±11.3	0.07^2^
**Male/female**	4/2	17/18	23/12	81/39	0.175^2^
**HBV/HCV/others**	0/0/0	11/27/5	4/21/9	63/33/26	-
**Stage 1/2/3/4/unknown**	-	-	-	48/48/16/4/4	-
**Grade 1/2/3/unknown**	-	-	-	18/74/23/5	-
**Mean size of tumor ± SD, cm**	-	-	-	5.31±3.69	-
**AFP levels, ng/mL, ≤20/>20/unknown**	-	-	-	62/53/5	-

1 4 of the 6 normal livers are “normal” liver tissues with concomitant cholangiocarcinoma.

2 Across all subjects (n = 196), age was analyzed by the Student *t* test and gender by Fisher's exact test.

AFP, alpha-fetoprotein; HBV, hepatitis B virus; HCC, hepatocellular carcinoma; HCV, hepatitis C virus; SD, standard deviation.

### Identification of the AFP-negative HCC by *mGSTP1*

**Figure 4:**
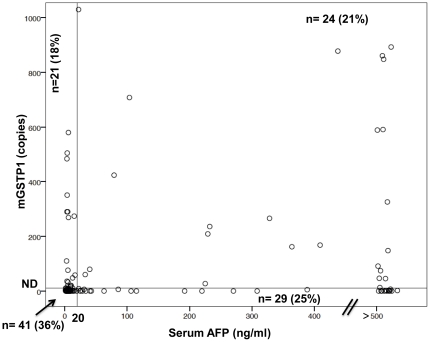
Scatter plot distribution of serum AFP levels (x-axis) and the amount of methylated 5′-end of the *GSTP1* DNA (*mGTSP1*) (y-axis) for 115 HCC samples. Each circle represents the value for an individual HCC case. A vertical reference line intersects at an AFP value of 20 ng/ml. A horizontal reference line intersects right above the MSP value of 0 as the reference for undetectable (ND), which is less than 10 copies per assay. The number of HCC cases and the percent of the total HCC in each of four areas are indicated.

As discussed earlier, the current “gold standard” serum marker, AFP and its fucosylated glycoform, L3, is of limited value because they have sensitivities of only 40% to 60% [3]. Moreover, there is currently no biochemical marker that can detect AFP-negative HCC, in which the serum AFP level is less than 20 ng/mL, as suggested by the American Association of Liver Diseases [3]. Because *mGSTP1* was previously detected in the blood of patients with HCC [16], [17], [18], [19], it was of interest to see whether we could detect *GSTP1* methylation in the HCC samples that were negative for AFP. We thus analyzed the incidence of *mGSTP1* in the HCC tissues for which AFP values were available (n = 115). We plotted the quantity of serum AFP on the x-axis and *mGSTP1* in tissue on the y-axis for each subject with HCC (Fig. 4). In this study population, 62 patients (53.9%, 62/115) with HCC had AFP serum levels less than 20 ng/mL and their HCC samples were therefore considered to be AFP-negative. Encouragingly, *mGSTP1* was found in 33.8% (21/62) of the AFP-negative HCC tissues, thus increasing the sensitivity of detecting HCC in tissues for which AFP values were available to us (n = 115) from 46.1% (53/115) with AFP alone to 64.3% (74/115) by combining two markers.

## Discussion

In this study, we observed that only the 5′ region of the 32 CpG sites examined (numbered −28 to +4 relative to the transcription start site) in the promoter region of the *GSTP1* gene that are methylated specifically in HCC compared to normal liver and hepatitis and cirrhotic liver tissues. On the other hand, CpG methylation was observed in the 3′ region (CpG sites from the −7 to +4) in most of the liver tissues studied including normal liver. We demonstrated that the MSP assay, designed for the 3′ region to analyze the *mGSTP1* promoter for distinguishing HCC from cirrhosis and hepatitis, would have poor specificity (60%). In contrast, a high specificity (97.1%) was obtained when the MSP assay was designed for the 5′ region of the promoter (Figure 3). These data not only prove our hypothesis that the location of the *GSTP1* DNA analyzed for methylation impacts the specificity for HCC as a biomarker for HCC detection but also provides an interpretation for the variation in HCC specificity of the *mGSTP1* promoter to distinguish HCC from other liver-disease tissues reported in previous publications, as examples listed in Table 1. As discussed, a recent review by von Vlodrop suggested the importance of the locations of CpG methylation in relation to gene expression and of the associations with clinicopathological characteristics in cancer [32]. For instance, only one of the three regions of the promoter CpG island of gremlin 1 was correlated with poor survival in clear cell renal carcinoma [35]. Together with the results of our recent studies, which showed that strand-biased methylation of the *APC* gene is present in liver and that methylation of only the sense strand is specific for HCC [33], these results highlight the importance of the locations of CpG methylation of a given marker for clinical applications, particularly in liver cancer, since both of the methylation markers we studied exhibited a liver-specific DNA methylation pattern when compared to the other 11 normal tissues examined.

We found significantly more detectable methylated CpG sites in adjacent non-HCC tissue compared to normal, hepatitis, and cirrhotic liver tissue using BS-PCR sequencing in the 5′ end region (p<0.0001 by Fisher's exact test), suggesting that methylation of the *GSTP1* promoter occurs early in the carcinogenetic process that results in HCC and that it plays a role in the cancer microenvironment or field effect. To date, such field effect biomarkers have been reported in several sites and organs, for example, head and neck, colon and rectum, prostate, breast, lung, esophagus, stomach, and skin, as reviewed by Chai 2009 [36], and also HCC [37], [38]. It has been suggested that the cancer microenvironment or the cancer field effect plays an important role in carcinogenesis [39] and that alterations in DNA methylation patterns may contribute to the field effect [40], [41]. Glutathione S-transferases are a family of enzymes that play an important role in detoxification and are responsible for protecting cells from cytotoxic and carcinogenic agents. Thus, one could easily understand that, if this detoxification enzyme is insufficiently expressed, the accumulation of carcinogens would promote carcinogenesis and give rise to a tumor in the field. It has been suggested that DNA methylation of the *GSTP1* gene suppresses the expression of *GSTP1* mRNA and protein [24], [25], [26]. Our data suggest that HCC-related DNA methylation does occur, although to a lesser extent in the adjacent non-HCC tissues, suggesting that methylation of the 5′ region of the *GSTP1* promoter could be part of the cancer microenvironment that cultivates the development of HCC. Because the methylation of the 3′ region of the promoter exists in the normal liver, it is possible that methylation of the 5′ region has a more profound effect on suppression of the *GSTP1* gene than originally thought. More study is needed to further understand the role of methylation of the 3′ region and 5′ region in the expression of the *GSTP1* gene.

As mentioned above, it has been suggested that promoter methylation of the *GSTP1* gene is a potential marker for HCC screening because this marker has been detected in the circulation of patients with HCC [16], [17], [18], [19] and could be a potential marker in combination with other markers for diagnosis and surveillance of persons at high risk for HCC. The methylation of the 5′ region was more specific (97.1% vs. 60%), but the sensitivity of detecting HCC was significantly higher if methylation of the 3′ region was analyzed (80% vs. 37.5%). As a result, the AUROC curves for both regions were similar (0.760 and 0.775). This finding suggests that HCC-related methylation occurs in the entire region of the promoter. Thus, when the extent of methylation was measured quantitatively, the increase in methylation was detected in either region regardless of the basal level of methylation. However, our study suggests that the assay to detect this marker should target the 5′ region of the promoter to obtain higher specificity, particularly when the end-point MSP is used.

Although this marker was found in only 37.5% of the HCC samples, the *mGSTP1* marker was detected in 33.8% of AFP-negative HCC in our study population. This result is important because no biochemical marker is currently available to detect AFP-negative HCC. Aberrant DNA methylation has already been suggested for use in a screening test to identify subjects that are at high risk for HCC. If one combines *mGSTP1* and serum AFP and detects the HCC specific region with the MSP assay, the sensitivity can be improved from 46.1% to 64.3%. Thus, *mGSTP1* could be a potential marker for HCC screening that would be complementary to AFP levels.

Using BS-PCR sequencing, we were able to detect different amounts of methylation at the 5′ region of the promoter in all 20 of the HCC samples and in 17/20 of adjacent non-HCC samples but limited amounts in hepatitis and cirrhosis tissue. Using the MSP assay, we targeted the 5′ region of the sense strand; methylation of the *GSTP1* gene was highly specific (97.1% specificity) even when compared to the adjacent non-HCC tissue. The BS-PCR sequencing data represent a collection of the methylation at that particular site from the templates that were amplified by a PCR reaction. This collection of methylation data does not represent individual molecules in the pool of DNA isolated from a given tissue. For a sample to be methylation positive using an MSP assay, all of the CpG sites included in the primers and probe would have to be methylated on one molecule except for limited miss-priming. Thus, in adjacent non-HCC samples, when we used BS-PCR sequencing and defined positive as detectable methylated CpG sites (low+high, Fig. 1C), 36.9% of CpG sites in the adjacent non-HCC samples were positive for 5′-end *mGSTP1* (Table 3); in contrast, with the MSP assay, only 4 of 120 (3.3%) were positive for the 5′-end. For HCC, with the BS-PCR sequencing assay, 65% of CpG sites in the HCC samples were positive for 5′-end *mGSTP1* (Table 3); only 45 of 120 (37.5%) were positive when measured by the 5′-end MSP assay. Nevertheless, the data from both assays suggest a higher density of methylation in the HCC samples compared to adjacent non-HCC samples.

In this study, we used different detection technologies for two MSP assays: TaqMan for the 5′-end MSP and SYBR Green for the 3′-end MSP. It is possible the difference of the sensitivity and specificity obtain by the these two methods is due to different detection technologies because that the 5′-end MSP could detect only the DNA molecules that contained methylated CpG sites in both primers and TaqMan probe locus, but the 3′-end MSP only tested CpG sites in the primers thus it is a less conservative assay. However, this concern should not affect the conclusion of this study that methylation of 5′-end region is more specific than that of the 3′-end region for detecting HCC from other liver disease tissues because this conclusion is also suggested by BS-CR sequencing analysis (Fig. 1). Furthermore, the low (48%) - to none (0%) specificity of the 3′-end methylation were observed by previous studies as we have referenced in Table 1 (study #4 [42] and study #5 [15]) in which the TaqMan was used in [15] and pyrosequencing was used in [42].

Interestingly, although most of the methylation detected in the *GSTP1* promoter region analyzed is symmetrical, some nonsymmetrical methylation was detected by BS-PCR sequencing (Fig. 1B). As discussed earlier, the BS-PCR sequencing data represent methylated molecules collected from the templates at the particular site and therefore do not represent individual molecule in the pool of DNA isolated from a given tissue. Analysis by the BS-PCR cloning sequencing will be needed to determine whether the nonsymmetrical methylation observed is multiple, continuous nonsymmetrical CpG site methylation on a molecule, as we discovered in the *APC* gene [33], or a collection from many templates, each of which contains 1 to 2 sparsely distributed nonsymmetrical methylated CpG sites that occurred because of errors of methylation. Nevertheless, most of the methylation in the *GSTP1* gene analyzed is symmetrical.

When we analyzed the different clinicopathologies (Table 5), we did not find a significantly higher incidence of *mGSTP1* in any of the HCC subsets (p>0.5 by the Kruskal-Wallis test). It has been suggested that infection with hepatitis B virus (HBV) or hepatitis C virus (HCV) increases the aberrant methylation of tumor suppressor genes in HCC, including the *GSTP1* gene [8], [9], [10], [13], [15], [43], in HBV-infected HCC but not in non-HBV-infected HCC or in non-HCV-infected HCC. This discrepancy could be due to the differences in the clinicopathological characteristics of the HCC tumors used in the different studies.

**Table 5:** Aberrant methylation of the *GSTP1* in HCC stratified by clinicopathological characteristics.

Comparison of HCC samples (n = 120)	*P* value^1^
Stage	0.723
Grade	0.160
HBV –HCC (n = 63) vs. non-HBV-HCC (n = 57)	0.801
HCV-HCC (n = 33) vs. non-HCV-HCC (n = 87)	0.906
AFP levels (<20 ng/mL vs. ≥20 ng/mL)	0.056

1 Kruskal-Wallis test.

AFP, alpha-fetoprotein; HBV, hepatitis B virus; HCC, hepatocellular carcinoma; HCV, hepatitis C virus.

Cancer is a disease of the genome and epigenome; thus, detection of genetic and epigenetic changes underlying the development of HCC should aid in the unambiguous detection of tumors. Interestingly, two of the potential HCC epigenetic DNA markers we examined, methylation of the *APC* [33] and *GSTP1* genes, exhibit liver-specific methylation patterns, suggesting that, in the search for epigenetic DNA markers for detection of HCC, the methylation status of normal liver should be taken into consideration when developing a sensitive and specific assay for the detection of HCC.

## Materials and Methods

### Subjects

The HCC tissues and the matched adjacent non-HCC liver samples used in this study were obtained with written informed consent from patients who underwent radical resection at The National Cheng-Kung University Medical center in accordance with the guidelines of the National Cheng-Kung University institutional review board. Archived DNA samples (35 hepatitis and 35 cirrhosis) were obtained from the Buddhist Tzu Chi Medical Center, Hualien, Taiwan, in accordance with the Buddhist Tzu Chi Medical Center institutional review board protocols. DNA from normal liver (n = 4), esophageal, and colon tissues was obtained from The Johns Hopkins University School of Medicine in accordance with The Johns Hopkins University Institutional Review Board protocols. The Institutional Review Boards of Drexel University College of Medicine, the Buddhist Tzu Chi Medical Center, The Johns Hopkins University School of Medicine, and The National Cheng-Kung University Medical center specifically approved this study. Normal liver (n = 1), heart, and lung tissue samples obtained from the National Disease Research Interchange, Philadelphia were given to us by Immunotope, Inc (Doylestown, PA), and normal peripheral blood mononuclear cell DNA was obtained as a gift from the laboratory of Dr. Pooja Jain (Drexel University College of Medicine). One normal liver tissue DNA sample was purchased from Capital Biosciences (Rockville, MD), and stomach 1–4, pancreas, kidney, spleen, breast, brain, trigeminal ganglion, and fetal liver DNA was purchased from Biochain (Hayward, CA). The subject profile is listed in Table 2, Table 4 and Table S2.

### DNA isolation and bisulfite treatment

DNA was isolated using the Qiagen DNAeasy Blood and Tissue kit (Qiagen, Valencia, CA) according to the manufacturer's instructions. The DNA concentration was measured using a NanoDrop 1000 spectrophotometer (Thermo Fisher Scientific Inc, Wilmington, DE) at 260 nm absorbance. Bisulfite treatment was performed using Qiagen EpiTect Bisulfite conversion kits (Qiagen) following the guidelines of the manufacturer.

### Preparation of reconstituted standards of methylated and unmethylated DNA for BS-PCR sequencing and MSP assays

To determine the assay sensitivity of BS-PCR sequencing and MSP assays to detect methylated DNA and estimate the relative amount of methylated DNA in a given sample, we prepared a reconstituted sample set (*i.e.*, a known amount of methylated DNA in a background of unmethylated DNA). Bisulfite-converted human universal methylated DNA control (Zymo Research, Seattle, WA) was used as the methylated DNA standard. Bisulfite-treated DNA from normal human peripheral blood mononuclear cells that was confirmed by sequencing to be unmethylated in the *GSTP1* region of interest was used as a source of unmethylated DNA and was quantified by the BS-actin real-time PCR assay, which primers were designed within regions lacking CpG sites, so that CpG methylation status would not affect primer binding [33]. On the basis of quantification by BS-actin PCR, reconstituted sample sets were prepared in the following ratios: (1) 0% methylated DNA, 100% unmethylated DNA; (2) 10% methylated DNA, 90% unmethylated DNA; (3) 25% methylated DNA, 75% unmethylated DNA; (4) 50% methylated DNA, 50% unmethylated DNA; and (5) 100% methylated DNA.

### BS-PCR DNA Sequencing

Bisulfite specific primers were designed using Methyl Primer Express software (Life Technologies, Applied Biosystems, Foster City, CA) to amplify the promoter region of the *GSTP1* gene for both the sense and antisense strands; the primer sequences are described in Table S1. PCR was performed in an Eppendorf Mastercycler thermocycler for 40 cycles with hot-start Taq polymerase (Qiagen). The PCR program started with activation of the polymerase at 95°C for 15 min followed by denaturation at 95°C for 30 s, annealing at the respective annealing temperature (Table S1) for 30 s, and extension at 72°C for 30 s, followed by a final 4-min extension at 72°C and cooling at 4°C for all primer sets. The reaction was assembled in a final volume of 20 µl containing 0.5 U HotStart Taq (Qiagen), 1× PCR buffer, 200 µM of dNTPs, 0.5 µM of each primer, and bisulfite-treated DNA templates. PCR products were run on 1% agarose gel with 1× TAE buffer. The PCR product of the correct size was excised, and the gel was purified with Qiagen Gel Purification kit (Qiagen) and sent with the appropriate primer for sequencing to the NAPcore facility at the Children's Hospital of Philadelphia, Philadelphia, PA. Sequencing results were analyzed using ClustalW software (available at http://www.ch.embnet.org/), Chromas 2.3 software (Technelysium, Tewantin, Queensland, Australia), and Finch TV version 1.4.0 (Geospiza Inc, Seattle, WA).

### MSP assays

A quantitative real-time methylation specific PCR (MSP) assay for the 5′-end region was developed with the primer pair and Taqman probe as shown in Table S1, and illustrated in Figure 3. More specifically, this is a MethyLight assay since a TaqMan probe was included in addition to methylation specific primers used in the assay. This 5′-end MethyLight assay was referred as 5′-end methylation specific PCR (MSP) assay. For the 5′-end MSP, A 10-µl reaction was assembled using the Roche Light Cycler 480 Real-Time PCR system (Roche Applied Science, Mannheim, Germany). The reaction contained a 1× LightCycler 480 Probes Master, 1.0 µM primers, 0.2 µM probe, and the DNA template. The PCR reaction was performed under the following conditions: 95°C 10 min, (95°C 10 s, 65°C 30 s, 72°C 10 s)×50 cycles, 40°C 30 s. The MSP assay for the 3′-end region used in the study was modified from the method previously described [15] by using the LightCycler 480 SYBR Green I Master. For the 3′-end MSP, A 10-µl reaction was assembled using the LightCycler 480 SYBR Green I Master (Roche Applied Science, Mannheim, Germany). The reaction contained 1× LightCycler 480 SYBR Green I Master, 1.0 µM primers, and the DNA template. The PCR reaction was performed under the following conditions: 95°C 10 min, (95°C 10 s, 60°C 15 s, 72°C 10 s)×45 cycles, melt curve analysis (95°C 5 s, 65°C 60 s, 97°C), 40°C 30 s.

### Statistical Analysis

To test whether age and gender were evenly distributed across both HCC and non-HCC groups, the Student *t* test was performed for age and Fisher's exact test was performed for gender. To study the distribution of *GSTP1* 5′-end MSP values in HCC tissues across the categories of stage, grade, HBV status, HCV status, and AFP groups (<20 or >20 ng/ml), a Kruskal-Wallis test was performed. Stages 2, 3, and 4 were combined into one group, and Grades 2 and 3 were combined into one group because the numbers of samples in stage 3 (n = 3), stage 4 (n = 1), and grade 3 (n = 9) were low. ROC curves, areas under the ROC curves, comparisons between ROCs and the scatter plot distribution of serum AFP levels (y-axis) versus the amount of *mGSTP1* DNA distribution was constructed using the PASW software (IBM, New York).

## Supporting Information

Figure S1 **The representative chromatograms of BSP sequencing of the reconstituted standards: 0% methylated+100% unmethylated DNA (0%);10% methylated DNA+90% unmethylated DNA (10%); 25% methylated DNA+75% unmethylated DNA (25%); 50% methylated DNA+50% unmethylated DNA (50%); and 100% methylated DNA (100%), from both sense (GSTP1_S_F/R) and antisense (GSTP1_AS_F/R) bisulfite specific PCR sequencing primers, as indicated.** The boxed areas are the areas of the examples showing the relative “C” and “T” peaks in the chromatogram from each sample of the reconstituted standards by each primer set as indicated.
(TIF)

Figure S2 **Amplification and standard curves of the 5′-end (A) and 3′-end (B) MSP assays.** Various concentrations of human methylated bisulfite-converted genomic DNA reconstituted in unmethylated DNA controls, as indicated, were amplified by the *GSTP1* MSP assays as detailed in Materials and Methods. The curves generated by different amounts of input DNA (copies) per reaction are indicated.
(TIF)

Table S1 (DOCX)

Table S2 (DOCX)
