# Supplementary figures and images for: Impact of the Location of CpG Methylation within the GSTP1 Gene on Its Specificity as a DNA Marker for Hepatocellular Carcinoma
Source: PLoS One. 2012 Apr 20;7(4):e35789. doi: 10.1371/journal.pone.0035789 (PMC3335004; doi:10.1371/journal.pone.0035789)

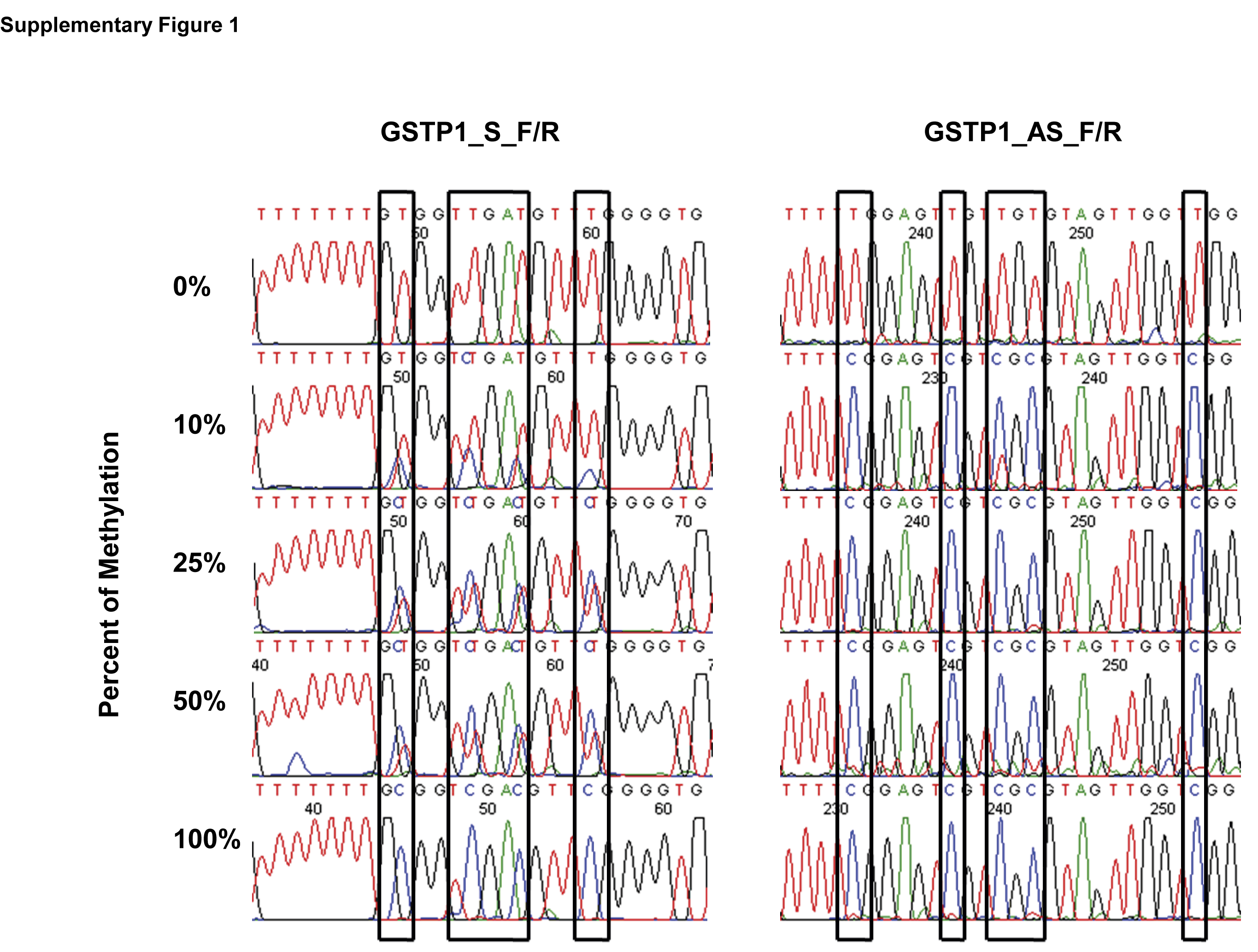

Supplement: Figure S1 — The representative chromatograms of BSP sequencing of the reconstituted standards: 0% methylated+100% unmethylated DNA (0%);10% methylated DNA+90% unmethylated DNA (10%); 25% methylated DNA+75% unmethylated DNA (25%); 50% methylated DNA+50% unmethylated DNA (50%); and 100% methylated DNA (100%), from both sense (GSTP1_S_F/R) and antisense (GSTP1_AS_F/R) bisulfite specific PCR sequencing primers, as indicated. The boxed areas are the areas of the examples showing the relative “C” and “T” peaks in the chromatogram from each sample of the reconstituted standards by each primer set as indicated. (TIF) [file pone.0035789.s001.tif]

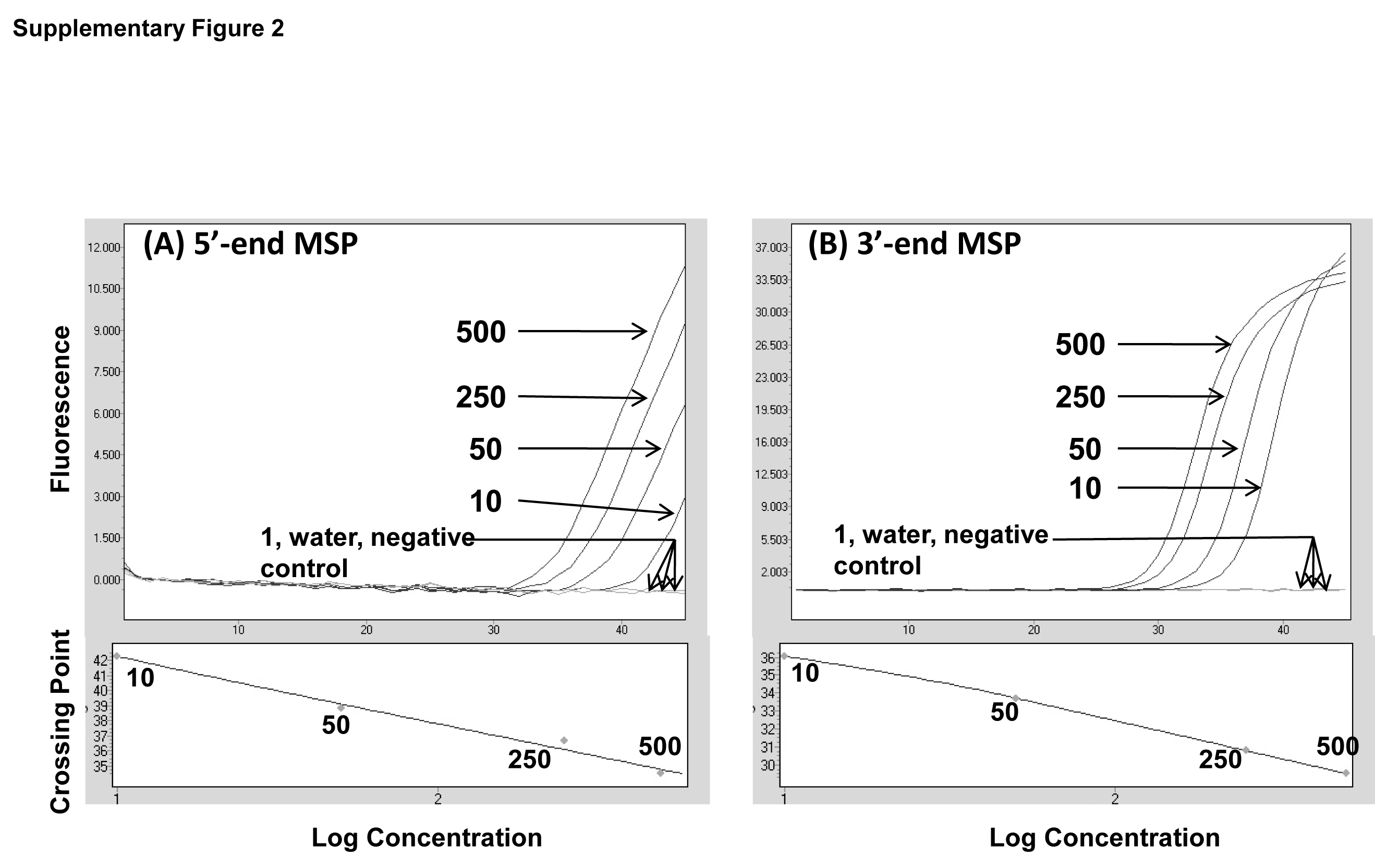

Supplement: Figure S2 — Amplification and standard curves of the 5′-end (A) and 3′-end (B) MSP assays. Various concentrations of human methylated bisulfite-converted genomic DNA reconstituted in unmethylated DNA controls, as indicated, were amplified by the GSTP1 MSP assays as detailed in Materials and Methods. The curves generated by different amounts of input DNA (copies) per reaction are indicated. (TIF) [file pone.0035789.s002.tif]
