# Supplementary material for: Impact of the Location of CpG Methylation within the GSTP1 Gene on Its Specificity as a DNA Marker for Hepatocellular Carcinoma
Source: PLoS One. 2012 Apr 20;7(4):e35789. doi: 10.1371/journal.pone.0035789 (PMC3335004; doi:10.1371/journal.pone.0035789)
Supplement: Table S1 — (DOCX) [file pone.0035789.s003.docx]

**Supplementary Table S1.** Primer and probe sequences used for bisulfite DNA sequencing and methylation-specific PCR assays for *GSTP1* (Genbank accession #M24485)

| **PCR Assay** | | **Primers and Probe Sequence** | **Annealing (^o^C)** | **Location** |
| --- | --- | --- | --- | --- |
| BSP | Sense | GSTP1_S_F: gggatttgggaaagagggaaa | 57.5 | 999-1409 |
|  |  | GSTP1_S_R: aacrtcctaaatcccctaaacc |  |  |
|  | Antisense | GSTP1_AS_F: ggttttatgttgggagttttga | 52 | 975-1310 |
|  |  | GSTP1_AS_R: tactccctaaaccccrcta |  |  |
| MSP | 5’-end | F: agttgcgcggcgatttc | 65 | 1034-1149 |
|  |  | R: ccgaccgctcttctaa |  |  |
|  |  | Probe: FAM-cggtcgacgttcggggtgtagcg-BHQ1 |  |  |
|  | 3’-end [15] | F: tcggaggtcgcgaggtt | 60 | 1200-1265 |
|  |  | R: cgcgcgtactcactaataacga |  |  |

AS, antisense; BSP, bisulfite-specific PCR; *GSTP1*, glutathione S-transferase π-1; MSP, methylation-specific PCR; PCR, polymerase chain reaction; S, sense; F, forward primer; R, reverse primer
