# Supplementary material for: Impact of the Location of CpG Methylation within the GSTP1 Gene on Its Specificity as a DNA Marker for Hepatocellular Carcinoma
Source: PLoS One. 2012 Apr 20;7(4):e35789. doi: 10.1371/journal.pone.0035789 (PMC3335004; doi:10.1371/journal.pone.0035789)
Supplement: Table S2 — (DOCX) [file pone.0035789.s004.docx]

**Supplementary Table S2**. Subject information for nonliver tissues and fetal liver

| Subject | Gender | Age, years | Pathological profile | HBV-infected | HCV-  infected |
| --- | --- | --- | --- | --- | --- |
| Pancreas | M | 27 | Normal | NA | NA |
| Kidney | F | 63 | Normal | NA | NA |
| Spleen | F | 83 | Normal | NA | NA |
| Stomach 1 | M | 27 | Normal | NA | NA |
| Stomach 2 | M | 50 | Normal | NA | NA |
| Stomach 3 | M | 58 | Normal | NA | NA |
| Stomach 4 | M | 29 | Normal | NA | NA |
| Lung | F | 50 | Normal | NA | NA |
| Heart | M | 67 | Normal | NA | NA |
| Colon 1 | F | 85 | Normal | NA | NA |
| Colon 2 | F | 77 | Normal | NA | NA |
| Esophagus | M | 73 | Normal | NA | NA |
| Trigeminal ganglion | M | 75 | Normal | NA | NA |
| Breast 1 | F | 78 | Normal | NA | NA |
| Breast 2 | F | 21 | Normal | NA | NA |
| Fetal liver | M | 29 weeks | Normal | NA | NA |

F, female; HCC, hepatocellular carcinoma; M, male; NA, not applicable
